# Mycobacterial Nucleic Acids Modulate Host Innate Immune Responses

**Published:** 2024-04-11

**Authors:** Geetha Srikrishna, C Korin Bullen, William R Bishai

**Affiliations:** Department of Medicine, Johns Hopkins School of Medicine, Baltimore, USA

## About the Study

Tuberculosis (TB), caused by *Mycobacterium tuberculosis* (Mtb), is a leading cause of death worldwide. Host-pathogen interplay that has evolved over thousands of years enables the host to contain Mtb infection in a majority of affected individuals, but these processes also facilitate prolonged survival of the pathogen within the host. In the face of delayed onset of acquired immunity against Mtb, early innate immune responses are critical in determining whether the infection is contained or whether it progresses to active disease [1, 2]. Mtb employs an evolutionarily refined molecular arsenal to subvert host immune responses, enabling its persistence in cells of the monocyte lineage contained within organized granulomas. Failure of the host immune system to contain the mycobacteria within the granuloma leads to necrosis, active cavitary disease, and pathogen spread.

Host innate immune responses are initiated upon Mtb infection with the recognition of Mtb PAMP (Pathogen-Associated Molecular Pattern) molecules by germ line-encoded Pattern Recognition Receptors (PRRs) expressed by innate immune cells [1–3]. This recognition leads to activation of signaling pathways, cellular events, and innate immune responses that help to contain, but not clear the infection, the latter stemming from evasion and co-opting strategies deployed by the pathogen. Among the responses elicited by Mtb PAMPs, recent studies have unraveled Cytosolic Surveillance Pathways (CSP) activated by Mtb DNA and RNA.

Mtb DNA gains access to cytosolic sensors through the ESX-1 system that mediates limited permeabilization of the phagosomal membrane [4]. Cytosolic sensors such as cyclic GMP-AMP synthase (cGAS) and Interferon activated gene 204 (IFI204) recognize Mtb DNA and promote production of Type I Interferons (IFNs) through activation of STING (stimulator of IFN genes), by producing the STING ligand cGAMP, a Cyclic Dinucleotide (CDN) [4–7]. Type I IFN-driven responses constitute a distinct blood transcriptional signature in active human TB disease [8]. In addition, Mtb releases the CDN cyclic-di-Adenosine Monophosphate (c-di-AMP), which promotes Type I IFN expression through direct binding to STING and modulates the outcome of infection [9]. An Mtb strain overexpressing c-di-AMP enhanced macrophage autophagy and significant disease attenuation in mice [9]. One mechanism of Mtb subversion of the STING-mediated Type I IFN response is by production of a CDN Phosphodiesterase (CdnP) which degrades both host-derived cGAMP and bacterial derived c-di-AMP. Loss of Mtb CdnP attenuated pathogen virulence [10]. Cytosolic DNA is also recognized by the Absence of Melanoma 2 (AIM2) sensor, leading to inflammasome activation and production of interleukins IL-1β and IL-18 [11, 12]. The balance between host responses and microbial survival strategies controls the levels of CDN agonists of STING, Type I IFNs and IL-1β, and determines the fate of infection. Understanding this balance in host-pathogen interactions is important for designing effective vaccines against TB [13]. For example, BCG vaccination prevents extrapulmonary TB in children, but not pulmonary TB. In adults, its efficacy is highly variable. Preclinical studies show that BCG strains overexpressing STING agonist c-di-AMP shows enhanced protection than BCG-WT in models of TB [14]. This protection is attributed to trained immunity or reprogramming of myeloid cells, and presents a unique opportunity to improve the efficacy of BCG [15, 16].

Endosomal bacterial RNA is known to activate signaling pathways through recognition by TLRs (TLR3, TLR7 and TLR8) [3]. More recent studies provide evidence that cytosolic Mtb RNA also functions as an important PAMP contributing to innate immune responses following Mtb infection. Exosomes released from Mtb infected macrophages have been shown to contain mycobacterial RNA [17]. Release of Mtb RNA into the cytosol of host macrophages has been shown to be mediated through Mtb SecA2 and ESX-1 secretion systems [18]. It is likely that cytosolic Mtb RNA survives in the hostile milieu of macrophages long enough to activate immune responses. Further, recent examination of human TB tissue using RNAScope reveals the presence of Mtb RNA in the cytoplasm of host cells [19].

Mycobacterial cytosolic RNA is recognized by PRRs that include nucleotide-binding domain, leucine-rich-containing family, pyrin domain-containing-3 (NLRP3), the Nod-Like Receptors (NRLs), the RIG-I-Like Receptors (RLRs), and Oligoadenylate Synthetases (OAS) and protein kinase R (PKR) [1, 3]. RLRs consist of Retinoic Acid-Inducible Gene I (RIG-I), Melanoma-Differentiation-Associated Protein 5 (MDA5) and Laboratory of Genetics and Physiology 2 (LGP2). They are a well-conserved family of structurally-related proteins that feature a central ATPase containing DExD/H-box helicase domain and a C-terminal repressor domain both of which are involved in RNA binding. In the case of RIG-I and MDA5, following activation through RNA engagement, two tandem Caspase Activation and Recruitment Domains (CARD) interact with the adaptor Mitochondrial Antiviral Signaling Protein (MAVS) to mediate induction of NF-κB and IRFs. Despite their structural similarity, RLRs detect distinct RNA species. For example, while RIG-I recognizes ssRNA/short dsRNA, MDA5 binds to long dsRNA [20–22].

The RLRs are well known for their roles in mediating Type I IFN anti-viral immune responses to cytosolic viral RNAs, but more recent studies have unraveled their contribution to the recognition of and responses to cytosolic bacterial RNA. Increased expression of RLRs have been observed in Mtb-infected murine macrophages [23]. Studies by Cheng and Schorey showed that mycobacterial mRNAs polA and ppe11 are recognized by the cytosolic RNA sensor RIG-I, but not MDA5, leading to IFNβ expression that is MAVS/TBK1/and IRF7-dependent [18]. IRF7 expression itself is induced by initial cGAS/STING-dependent IFN-β production, strongly implying that a crosstalk exists between the host DNA and RNA signaling pathways in response to Mtb infection, that lead to both an early DNA-mediated expression and a late RNA-mediated expression of Type I IFNs.

In order to understand whether other RLRs are involved in promoting responses to different Mtb RNA species, we recently examined immune responses in macrophages transfected with total Mtb RNA, and found that Mtb RNA induced proinflammatory cytokine expression in an MDA5-dependent manner. Mtb infection not only induced MDA5 expression, but also elicited multimerization of MDA5, strongly implying activation of the receptor [24]. In IFNγ-primed macrophages, we found that MDA5 in fact promoted Mtb survival through a culmination of inflammasome activation, IL-1β production, and attenuation of autophagy [24]. Our findings uncovered a distinct role for MDA5 compared to RIG-I, and revealed a functional dichotomy in immune responses elicited by these structurally related RLRs. Which pathway dominates during active infection and which distinct Mtb RNA species are recognized by RIG-I versus MDA5 still need to be addressed.

## Conclusion

In summary, recent studies continue to unravel recognition of Mtb nucleic acids by the host innate immune system and the evasion and exploitation strategies employed by the pathogen to promote its survival. Since innate responses to Mtb are crucial in determining the progress and outcome of TB, it is important to seek clearer understanding of the early events in host-pathogen interplay in order to design effective vaccines and therapeutics.

## References

[1] Chai Q, Wang L, Liu CH, Ge B (2020) New insights into the evasion of host innate immunity by *Mycobacterium tuberculosis*. Cell Mol Immunol 17:901–913. 32728204 10.1038/s41423-020-0502-z PMC7608469

[2] Ravesloot-Chavez MM, van Dis E, Stanley SA (2021) The innate immune response to Mycobacterium tuberculosis infection. Annu Rev Immunol 39:611–637. 33637017 10.1146/annurev-immunol-093019-010426

[3] Burkert S, Schumann RR (2020) RNA sensing of Mycobacterium tuberculosis and its impact on TB vaccination strategies. Vaccines (Basel) 8:67. 32033104 10.3390/vaccines8010067 PMC7158685

[4] Manzanillo PS, Shiloh MU, Portnoy DA, Cox JS (2012) *Mycobacterium tuberculosis* activates the DNA-dependent cytosolic surveillance pathway within macrophages. Cell Host Microbe 11:469–480. 22607800 10.1016/j.chom.2012.03.007 PMC3662372

[5] Collins AC, Cai H, Li T, Franco LH, Li XD, (2015) Cyclic GMP-AMP synthase is an innate immune DNA sensor for Mycobacterium tuberculosis. Cell Host Microbe 17:820–828. 26048137 10.1016/j.chom.2015.05.005 PMC4499468

[6] Wassermann R, Gulen MF, Sala C, Perin SG, Lou Y, (2015) *Mycobacterium tuberculosis* differentially activates cGAS- and inflammasome-dependent intracellular immune responses through ESX-1. Cell Host Microbe 17:799–810. 26048138 10.1016/j.chom.2015.05.003

[7] Watson RO, Bell SL, MacDuff DA, Kimmey JM, Diner EJ, (2015) The cytosolic sensor cGAS detects Mycobacterium tuberculosis DNA to induce Type I interferons and activate autophagy. Cell Host Microbe 17:811–819. 26048136 10.1016/j.chom.2015.05.004 PMC4466081

[8] Berry MP, Graham CM, McNab FW, Xu Z, Bloch SA, (2010) An interferon-inducible neutrophil-driven blood transcriptional signature in human tuberculosis. Nature 466:973–977. 20725040 10.1038/nature09247 PMC3492754

[9] Dey B, Dey RJ, Cheung LS, Pokkali S, Guo H, (2015) A bacterial cyclic dinucleotide activates the cytosolic surveillance pathway and mediates innate resistance to tuberculosis. Nat Med 21:401–406. 25730264 10.1038/nm.3813 PMC4390473

[10] Dey RJ, Dey B, Zheng Y, Cheung LS, Zhou J, (2017) Inhibition of innate immune cytosolic surveillance by an *M. tuberculosis phosphodiesterase*. Nat Chem Biol 13:210–217. 28106876 10.1038/nchembio.2254

[11] Fernandes-Alnemri T, Yu JW, Datta P, Wu J, Alnemri ES (2009) AIM2 activates the inflammasome and cell death in response to cytoplasmic DNA. Nature 458:509–513. 19158676 10.1038/nature07710 PMC2862225

[12] Hornung V, Ablasser A, Charrel-Dennis M, Bauernfeind F, Horvath G, (2009) AIM2 recognizes cytosolic dsDNA and forms a caspase-1-activating inflammasome with ASC. Nature 458:514–518. 19158675 10.1038/nature07725 PMC2726264

[13] Moreira-Teixeira L, Mayer-Barber K, Sher A, O’Garra A (2018) Type I interferons in tuberculosis: Foe and occasionally friend. J Exp Med 215:1273–1285. 29666166 10.1084/jem.20180325 PMC5940272

[14] Dey RJ, Dey B, Singh AK, Praharaj M, Bishai W (2020) Bacillus calmette-guerin overexpressing an endogenous stimulator of interferon genes agonist provides enhanced protection against pulmonary tuberculosis. J Infect Dis 221:1048–1056. 30901058 10.1093/infdis/jiz116 PMC7931846

[15] Netea MG, Joosten LA, Latz E, Mills KH, Natoli G, (2016) Trained immunity: A program of innate immune memory in health and disease. Science 352:aaf1098. 27102489 10.1126/science.aaf1098 PMC5087274

[16] Sanchez-Ramon S, Conejero L, Netea MG, Sancho D, Palomares O, (2018) Trained immunity-based vaccines: A new paradigm for the development of broad-spectrum anti-infectious formulations. Front Immunol 9:2936. 30619296 10.3389/fimmu.2018.02936 PMC6304371

[17] Singh PP, Li L, Schorey JS (2015) Exosomal RNA from Mycobacterium tuberculosis-infected cells is functional in recipient macrophages. Traffic 16:555–571. 25753779 10.1111/tra.12278 PMC5735426

[18] Cheng Y, Schorey JS (2018) *Mycobacterium tuberculosis*-induced IFN-beta production requires cytosolic DNA and RNA sensing pathways. J Exp Med 215:2919–2935. 30337468 10.1084/jem.20180508 PMC6219742

[19] Nargan K, Naidoo T, Msimang M, Nadeem S, Wells G, (2023) Detection of *Mycobacterium tuberculosis* in human tissue *via* RNA *in situ* hybridization. bioRxiv.

[20] Sha W, Mitoma H, Hanabuchi S, Bao M, Weng L, (2014) Human NLRP3 inflammasome senses multiple types of bacterial RNAs. Proc Natl Acad Sci U S A 111:16059–16064. 25355909 10.1073/pnas.1412487111 PMC4234566

[21] Keestra-Gounder AM, Tsolis RM (2017) NOD1 and NOD2: beyond peptidoglycan sensing. Trends Immunol 38:758–767. 28823510 10.1016/j.it.2017.07.004 PMC5624830

[22] Schlee M (2013) Master sensors of pathogenic RNA - RIG-I like receptors. Immunobiology 218:1322–1335. 23896194 10.1016/j.imbio.2013.06.007 PMC7114584

[23] Andreu N, Phelan J, de Sessions PF, Cliff JM, Clark TG, (2017) Primary macrophages and J774 cells respond differently to infection with *Mycobacterium tuberculosis*. Sci Rep 7:42225. 28176867 10.1038/srep42225 PMC5296737

[24] Bullen CK, Singh AK, Krug S, Lun S, Thakur P, (2023) MDA5 RNA-sensing pathway activation by *Mycobacterium tuberculosis* promotes innate immune subversion and pathogen survival. JCI Insight 8(20). 10.1172/jci.insight.166242 PMC10619499 37725440

